# ASIC1a-associated mechanical hypersensitivity in the GlaKO Fabry disease mouse model

**DOI:** 10.1016/j.ynpai.2025.100189

**Published:** 2025-06-26

**Authors:** Mayra Micaela Montes, Libia Catalina Salinas Castellanos, Georgina Oriana Mingolo Malnati, Juan Santiago Guidobono, Ariel Félix Gualtieri, Mariela Lacave, Romina De Lucca, María Natalia Gobetto, Pablo Gabriel Vetta, Zaira Soledad Verónica Náguila, Fernanda Toledo, Osvaldo Daniel Uchitel, Carina Weissmann

**Affiliations:** a Insituto de Fisiología Biología Molecular y Neurociencias-IFIBYNE-UBA-CONICET, LFBM, Argentina; b Instituto de Ecología, Genética y Evolución de Buenos Aires (IEGEBA), CONICET, UBA, Buenos Aires, Argentina; c Universidad de Buenos Aires, Facultad de Odontología, Cátedra de Odontología Legal, Forense e Historia de la Odontología, Argentina; d Universidad de Buenos Aires, Facultad de Odontología. Cátedra de Histología y Embriología. Buenos Aires, Argentina; e PGV Labs, Buenos Aires, Argentina; f Bioterio Central FCEN, Buenos Aires University, Argentina

**Keywords:** Fabry disease, Chronic pain, ASIC1a, Mechanical sensitivity, Thermal sensitivity

## Abstract

Different lines of evidence point to a role for Acid-sensing ion channel 1 (ASIC1) in pain perception, acting as sensors in both the central nervous system and peripheral tissues. While elevated ASIC1 protein expression has been documented in various pain conditions, our study focuses on its involvement in the context of Fabry disease (FD).

Using a mouse model of FD, we observed a significant increase in ASIC1 protein expression in pain-related areas including the anterior cingulate cortex (ACC), as well as the spinal cord (SC) and dorsal root ganglia (DRG) at the lumbar, thoracic, and cervical levels. This upregulation was accompanied by increased ASIC1a mRNA levels and ERK phosphorylation. Moreover, in FD mice, ASIC1 protein expression was found to be modulated by age and sex: it was higher in female mice than in males, and increased with age in both sexes.

These findings, together with our previous work showing unaltered ASIC1a mRNA levels but microRNA-mediated regulation of ASIC1a protein in the formalin-induced acute pain model, highlight distinct mechanisms of ASIC1a regulation in FD-associated versus acute pain. Additionally, our study revealed heightened mechanical sensitivity in FD mice that could be prevented using a channel blocker, further highlighting the involvement of ASIC1a channels in pain pathways associated with Fabry disease. Our findings suggest that ASIC1a channels may serve as promising therapeutic targets for pain management in Fabry disease.

## Highlights

• ASIC1 and ASIC1a mRNA levels are upregulated in pain-related regions in a Fabry disease (FD) mouse model.
• Elevated phospho-ERK signaling is detected in the nervous system of FD mice.
• Age-dependent changes in ASIC1 expression and mechanical hypersensitivity are observed in FD mice.
• ASIC1a inhibition prevents heightened mechanical sensitivity in FD mice.

## Graphical abstract

**Figure:**
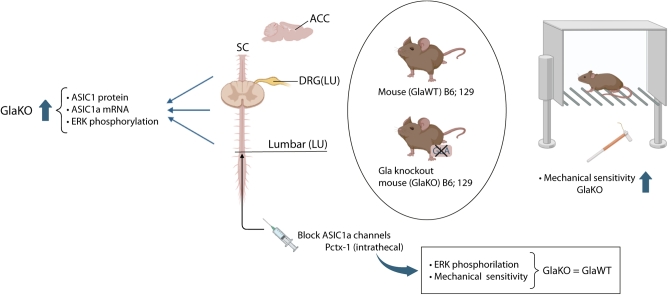
**Schematic representation of the main findings.** GlaKO mice (Fabry disease model with *Gla* gene knockout on a B6;129 background) exhibited increased ASIC1 protein, ASIC1a mRNA expression, and ERK phosphorylation in key pain-related regions: the anterior cingulate cortex (ACC), lumbar dorsal root ganglia (DRG), and lumbar spinal cord (SC). These molecular changes were accompanied by heightened mechanical sensitivity, assessed using the von Frey test. Intrathecal administration of PcTx-1, an ASIC1a channel blocker, normalized ERK phosphorylation and mechanical sensitivity, restoring GlaKO values to those of GlaWT controls.

## Introduction

Pain perception is a complex physiological process involving various molecular players including ion channels. Fabry disease, a lysosomal storage disorder, is caused by the absence or reduction in the activity of the alpha galactosidase A enzyme (GLA) that leads to the accumulation of glycosphingolipids (GS), in particular Globotriaosylceramide (Gb3) and a derivative, globotriaosylsphingosine (lysoGb3) (Germain, 2010). Over time, this lipid accumulation leads to loss of sensory nerve fibers (small fiber neuropathy), sensory abnormalities and pain, as well as organ dysfunction including myocardial infarction, arrhythmia, renal dysfunction, and cerebrovascular events such as strokes (Burand and Stucky, 2021). Additionally, the inflammatory response is continuously being activated further exacerbating the process (Rozenfeld and Feriozzi, 2017).

Neuropathic pain and pain crisis are early manifestations in FD (Politei et al., 2016). Despite different pharmacological approaches, antiepileptics, non-steroidal anti-inflammatory drugs, and opioids, pain treatment in FD patients is a problem that has not yet been solved. Although enzyme replacement therapy may reduce pain, it does not completely resolve it (Politei et al., 2016).

Different ion channels have been associated with FD (Weissmann et al., 2021). Evidence pointing to a role for Acid sensing ion channels (ASICs), and ASIC1a channels in particular, in pain (Wu et al., 2004, Duan et al., 2007, Deval et al., 2015, Voilley et al., 2001), along with the involvement of calcium channels in inflammation (Duzhyy et al., 2021) led us to propose a role for ASIC1s in pain in FD (Weissmann et al., 2021). ASIC proteins are involved in neurosensory mechanotransduction in mammals (Chen and Wong, 2013) and are composed of different ASIC subunits (ASIC1a, ASIC1b, ASIC2a, ASIC2b, ASIC3 and ASIC4) that can combine into hetero- or homotrimeric channels varying in their location and proton sensitivity (Vullo and Kellenberger, 2018).

Psalmotoxin-1 (PcTx-1), a polypeptide toxin isolated from the venom of the tarantula *Psalmopoeus cambridgei* specifically inhibits homomeric ASIC1a with an IC50 of ∼ 1 nM and no other ASIC subtypes or voltage-gated ion channels (Gründer and Chen, 2010). Pctx-1 has been shown to prevent acute pain (Mazzuca et al., 2007, Bohlen et al., 2012, Gobetto et al., 2022), as well as pain from chronic pain models decreasing both, thermal and mechanical sensitivity (Mazzuca et al., 2007).

We showed previously in *in vitro* experiments that the presence of accumulated Gb3 or lysoGb3 leads to the upregulation of ASIC1a channels (Salinas et al., 2020). Although the mechanism is still far from solved, GS, directly or indirectly, in vitro act increasing ASIC1a RNA levels (Salinas et al., 2020). Moreover, this upregulation leads to ERK phosphorylation (Salinas et al., 2020) consistent with reports on ERK activation in the CNS and PNS in pain conditions (Qu et al., 2016; 2016., Alvarez et al., 2009, Ji et al., 2009, Ji et al., 1999, Pang et al., 2008, Choi et al., 2015). Additionally, in an in vivo acute pain mouse model (formalin injection), where ASIC1a inhibitors have been shown to reduce pain responses (Gobetto et al., 2022), we detected increased ASIC1 protein expression in specific tissues along the pain pathway (Mazzuca et al., 2007).

In the present study, we extended our investigation to assess ASIC1 protein levels in a mouse model of FD which lacks GLA, as established by Ohshima et al (Ohshima et al., 1997). Consequently, we tested if ASIC1a blockers could affect mechanical sensitivity.

Throughout this manuscript, the terms ASIC1 and ASIC1a are used depending on the specificity of the experimental approach. ASIC1a is a specific isoform of the ASIC1 channel; however, due to the lack of isoform-specific antibodies, protein-level detection does not allow for isoform distinction and is thus referred to as ASIC1. In contrast, when using mRNA analysis or pharmacological agents that specifically target ASIC1a, the term ASIC1a is used.

Our findings demonstrate that ASIC1 protein levels are elevated in regions of the pain pathway in GlaKO mice, similar to the formalin mouse model. However, unlike in the acute pain model (formalin injection), where ASIC1 protein levels increase but ASIC1a mRNA remains unaltered, GlaKO mice exhibit both an increase in ASIC1 protein levels and a sustained elevation in ASIC1a mRNA over time.

## Materials and methods

### Animal model

All experiments involving animals were approved by the Institutional Committee for the Care and Use of Laboratory Animals (CICUAL) of the Faculty of Exact and Natural Sciences, University of Buenos Aires, and conducted in accordance with national ethical guidelines. Male and female mice aged 4 and 8 months (n = 6–14 per group) were used. In this study, we refer to B6129SF2/J animals as GlaWT, and to B6;129-Glatm1Kul/J animals (JAX #003535) as GlaKO, both strains commonly used in Fabry disease (FD) research. The GlaWT strain corresponds to the genetic background recommended by Jackson Laboratory to serve as the control for the GlaKO line. To maintain a consistent genetic background and avoid confounding effects of mixed strains, each line was bred in-house through homozygous mating (GlaWT × GlaWT and GlaKO × GlaKO), and wild-type animals were not littermates of knockouts. Given that Gla is located on the X chromosome, male animals are hemizygous (WT or KO). In contrast, heterozygous females exhibit variable gene expression due to random X-chromosome inactivation, resulting in mosaicism that can introduce phenotypic variability. To ensure consistent and clearly defined genotypes for comparative studies, we maintained separate homozygous lines for GlaWT and GlaKO, rather than using heterozygous females for breeding. This breeding strategy follows established protocols in previous studies using the same FD mouse model (Jabbarzadeh-Tabrizi et al., 2020), including investigations of background strain effects. No further backcrossing to C57BL/6 or 129 substrains was performed. Both colonies have been bred in-house since 2019 and were at generations N6 to N8 at the time of the experiments. The mice were housed in groups of 4 per cage on a 12 h:12 h light–dark cycle at climate-controlled rooms with a constant room temperature of 22 ± 1◦C and in conditions of relative humidity of 65 % and provided with food and water ad libitum. Mice were randomly divided into groups depending on the experimental design. Previous to any animal testing, we confirmed that mice presented a normal behavior, using supervised protocols (codes 112, 131), approved by the CICUAL Experimentation Ethical Committee, indicating that mice had a healthy status. ARRIVE reporting guidelines were used (du Sert et al., 2020). While breeding schemes involving heterozygous females are sometimes proposed to generate both WT and KO offspring in the same litter, this approach is not feasible for generating WT and KO male littermates in X-linked models like Gla. To obtain both genotypes, an additional colony of WT males must be maintained for mating with heterozygous females. As a result, the WT males obtained from this scheme are not true littermates of the KO males, defeating the intended purpose. For this reason, and to ensure consistent and clearly defined genotypes, we used separate colonies for GlaWT and GlaKO mice.

### Behavioural tests

In all analyses, a single animal constituted the experimental unit. Data collection and recording were conducted by an experimenter blinded to the treatment conditions. The number of animals used in each group was as follows: Male animals: 4 months (4 M) GlaWT: 28; GlaKO: 36; 8 months (8 M) GlaWT: 19; GlaKO: 22. Female animals: 4 M GlaWT: 22; GlaKO: 29; 8 M GlaWT: 18; GlaKO: 29. The sample size was determined based on preliminary analyses and pilot experiments, which assessed the initial variability in mechanical sensitivity responses. These pilot studies helped estimate the necessary number of animals to achieve reliable detection of differences between groups. Additional animals were required to account for the effects of different drug treatments used. Animals with a mechanical sensitivity resulting in a 50 % withdrawal threshold below 0.09 were excluded from the analysis, as this fell outside the range of acceptable baseline thresholds.

### Mechanical sensitivity

Mechanical withdrawal thresholds were determined using the von-Frey test based on the up-and-down-method (Chaplan et al., 1994). Mice within acrylic glass boxes were placed on a wire mesh. Habituation for 60 min was performed one day before the experiment. The day of the experiment, the animals were placed on the mesh and the plantar surface of the hind paw was touched with a von-Frey filament from 0,04 to 2,0g (North Coast Medical Von Frey Filaments). Upon paw withdrawal the next thinner von-Frey filament was applied. If no paw withdrawal was observed, the next thicker von-Frey filament was used. Each hind paw was tested 3 times. The 50 % withdrawal threshold was calculated using the method and open software described by Cano et al (Gonzalez-Cano et al., 2018)**.**

### Thermal sensitivity

#### Hot plate test

Each mouse was placed within acrylic glass boxes on top of a thermo-regulated heated plate (PGV Labs). Habituation to the hot plate was performed for 60 min after habituation to the Von-Frey setting one day prior to the experiment. The day of the experiment, the plate was set at 55℃ ± 0.1℃. The time (in seconds) between the placement of the animal and the first response: paw licking/jumping was measured as latency in seconds. A 30-s cut-off was used to prevent tissue damage.

#### Treatments

Animals treated with Pctx-1 or PBS were injected 30 min before the tests.

For this purpose, animals received an intrathecal injection of 10 µL of Psalmotoxin-1 0.1 nmol (PcTx-1, Alomone Labs) or vehicle (PBS) using a Hamilton syringe with a 30-gauge needle under isoflurane inhalation delivered through a face-snout mask.

PcTx-1 was dissolved on the day of experimentation in PBS. BSA (1 mg/mL) was added to the solution of PcTx-1 to prevent non-specific adsorption of the toxin.

Intrathecal injections were performed under light isoflurane anesthesia between spinal segments L5 and L6 according to the method described by Hyden and Wilcox for mice (Hylden and Wilcox, 1980). All experimental results were collected and registered by an experimenter blinded to the treatment.

#### Tissue collection

Micre were sacrificed under deep anesthesia induced by intraperitoneal injection of avertin (2% Tribromoethanol, SIGMA) at the end of the behavioral assessment. ACC; SC and DRG thoracic (THO), lumbar (LU) and cervical (CE) segments were dissected and separated for western blot and RT-PCR analysis. The tissue was shock-frozen in liquid nitrogen for storage at − 80°C before further processing. For ACC and SC, an n = 4/5 per group was used. In the case of DRGs, a pool of at least 3 DRGs corresponding to the same segment was used per experiment to detect a signal**.**

#### Protein analysis

For protein analysis, tissues were processed using a manual homogenizer with protease inhibitors. The suspension was centrifuged for 10 min at 10,000 rpm and the supernatant was used with loading buffer 6x in the electrophoresis run as described before (Salinas et al., 2020). Protein concentration of all samples was determined using a NanoDrop spectrophotometer (Thermo Scientific), and 20 μg of total protein lysate was loaded per lane for all experiments. No differences in tubulin levels were observed between genotypes or between 4- and 8-month-old mice (see Supplementary Fig. S3 and main figures showing tubulin levels for the same amount of loaded protein), validating tubulin as a loading control. The following primary antibodies were used: rabbit polyclonal antiASIC1 (Alomone ASC-014, 1:1000); mouse monoclonal anti-tubulin (DM1a; Cell signaling #3873, 1:5000); rabbit polyclonal anti-total ERK (Santa Cruz, C9, 1:500); rabbit polyclonal anti phospho-ERK (Cell Signaling, SC-7383, 1:500). Reactive bands were detected by the LI-COR Odyssey system, using secondary antibodies: 926–68073 IRDye 680RD Donkey anti-Rabbit IgG or 926–32212 IRDye 800CW Donkey anti-Mouse. Images were taken using the LI-COR Odyssey system and quantified with ImageJ software (NIH, Bethesda, MD, USA).

#### Quantitative PCR

Ribonucleic acid (RNA) samples were prepared from tissue using Trizol reagent (Thermo Fisher, Waltham, MA, USA) according to manufacturer guidelines. Then, 200 ng of total RNA was subjected to reverse transcription using the iScript™ cDNA Synthesis Kit—Bio-Rad to obtain 10 ng/mL cDNA. Next, the real-time polymerase chain reaction (PCR) analysis was performed based on the SYBR Green PCR Master Mix from the Biorad assay with the StepOne Real-Time PCR System (Life Technologies, Carlsbad, CA, USA). The ASIC1a primers used were those used by Papalampropoulou-Tsiridou et al. (Wu et al., 2004). Primers to amplify miR-485-5p were those used by Gao et al. (Gao et al., 2019). The expression of the gene of interest was normalized using GAPDH as a housekeeping gene as used in [61]. To avoid differences due to possible RNA degradation or different reverse transcription efficacy, the relative expression levels were calculated using the Ct method.

#### Immunohistochemistry

Mice were perfused with 4 % Paraformaldehyde and brains were sectioned and stained as previously performed (Gatto et al., 2020). Cryosections (20 µm) adhered to slides from blocks embedded in OCT were obtained and stained with antibodies as described before (Gatto and Weissmann, 2018). Primary antibodies used were Gb3 (TCI, #A2506; 1:100); mouse monoclonal anti-tubulin (DM1a; Cell signaling #3873, 1:3000); rabbit polyclonal antiASIC1 (Alomone ASC-014, 1:100) and Goat Alexa 488 anti-mouse (1:500); Goat Alexa 488 anti-Rabbit (1:500) and DAPI used to label nuclei.

### Data analysis and figure Preparation

Statistical analyses were performed using either Student’s *t*-test or ANOVA, followed by Tukey’s post hoc tests where appropriate, depending on the specific hypothesis being tested. Prior to these analyses, data distributions were evaluated for normality using the Shapiro-Wilk test. For behavioral analysis in Fig. 3, a generalized linear model (GLM) with Log-Normal error structure, and a log-link function, was performed for both mechanical and thermal sensitivity. For treatment analysis (Fig. 4), the statistical analysis was performed using a linear mixed-effects model (a mean comparison analysis, similar to the traditional ANOVA, that use the maximum likelihood estimation method to estimates the parameters of the analysis and include the effect of random explanatory variables), including additionally a constant variance function structure to explain the variance heterogeneity, crossing treatment with genotype. Tukey post-hoc tests were used in all cases. Details of the statistical methods applied are provided in each figure legend. Data visualization and plotting were conducted using Graph Pad Prism (version 8; Graph Pad Software Inc., La Jolla, CA, USA), and R software (R core Team 2025, https://www.R-project.org/) using the integrated development environment RStudio (Posit team 2025, URL https://www.posit.co/.). Figures and illustrations were created with Adobe Illustrator (version 10; Adobe Inc.).

## Results

### Changes in ASIC1 expression levels in Pain-Related areas of a Fabry mouse model

In our previous studies, we demonstrated that ASIC1a inhibitors (Mazzuca et al., 2007) reduced pain responses in a mouse model of acute pain induced by formalin (Gobetto et al., 2022). These experiments revealed an elevation in ASIC1 channels in several regions within the pain pathway. Additionally, our in vitro studies showed that the presence of Gb3 led to increased ASIC1a mRNA and protein levels (Salinas et al., 2020). Gb3 accumulation is well-documented in various regions of FD patients, particularly in the DRG and SC (Hofmann et al., 2018, Jabbarzadeh-Tabrizi et al., 2020). Although Gb3 presence in the brain is less extensively reported it has been detected in areas such as the anterior cingulate cortex (ACC) (Del Tredici et al., 2020, Kummer et al., 2018), as confirmed in our current study (Supplementary Fig. S1).

**Fig. 1:**
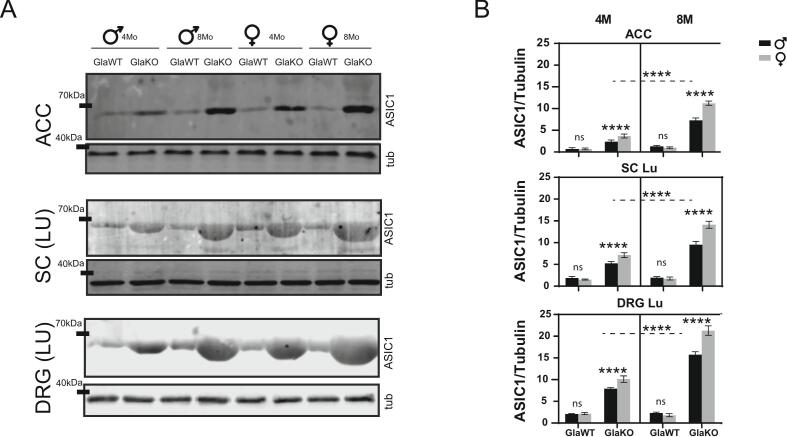
**ASIC1 protein expression levels in pain-related** areas of a Fabry mouse model A) Representative WB membrane showing ASIC1 expression in the ACC, lumbar SC, and lumbar DRG of 4-month old (4M) and 8-month-old (8M) GlaWT and GlaKO male and female mice. **B)** Quantitative plot of ASIC1/tubulin ratios comparing ACC, lumbar SC, and lumbar DRG in 4- and 8-month-old GlaWT and GlaKO mice. Data are presented as means ± SEM. Statistical analysis was performed using 3-way ANOVA followed by Tukey's post-hoc test; ****p < 0.0001; n = 5 per group.

In this study, we analyzed ASIC1 protein expression in a Fabry disease (FD) mouse model, examining both male and female animals at different ages (4 months vs. 8 months). We focused on the ACC, cervical, thoracic, and lumbar regions of the spinal cord (SC), and the dorsal root ganglia (DRG) within the pain pathway as previously analyzed (Gobetto et al., 2022). As shown in Fig. 1, ASIC1 protein expression was markedly higher in GlaKO mice compared to GlaWT controls across multiple regions of the pain pathway. In the ACC of 4-month-old males, ASIC1 protein levels were significantly elevated in GlaKO animals (2.39 ± 0.165) relative to GlaWT (0.697 ± 0.120), with an even more pronounced difference observed in females (3.672 ± 0.201 in GlaKO vs. 0.701 ± 0.096 in GlaWT). No differences were detected in the cerebellum, used as a control region (Supplementary Fig. S2). These genotype-dependent differences were more pronounced at 8 months, with ACC levels reaching 7.319 ± 0.234 in GlaKO males and 11.238 ± 0.215 in females. Similar patterns were observed in the spinal cord (SC) and dorsal root ganglia (DRG). In 4-month-old males, lumbar DRG levels were 7.881 ± 0.122 in GlaKO mice versus 2.056 ± 0.054 in GlaWT; by 8 months, these values rose to 15.745 ± 0.307 in males and 21.294 ± 0.490 in GlaKO females (Fig. 1).

**Fig. 2:**
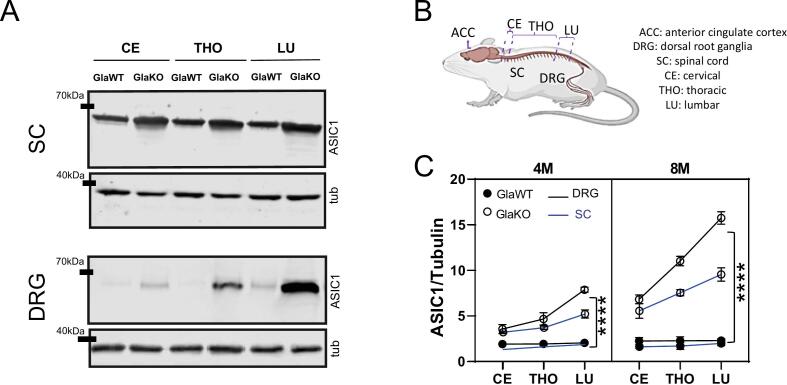
**Regional and age-dependent upregulation of ASIC1 protein expression along the pain pathway in GlaKO male mice A)** Representative WB membranes depicting ASIC1 protein expression in Anterior Cingular cortex (ACC), the spinal cord (SC) and dorsal root ganglia (DRG) from cervical, thoracic, and lumbar regions of 4-month-old (4M) GlaWT and GlaKO male mice**. B)** Schematic illustration of the anatomical regions analyzed, including their abbreviations. **C)** Quantitative plot of ASIC1/tubulin ratios in 4- and 8-month-old (8M) GlaWT and GlaKO male mice, illustrating a progressive increase in ASIC1 protein expression from cervical to lumbar regions in the SC (blue) and DRG (black). Lines connect points representing different SC or DRGs segments (CE, THO, LU), indicating the distribution across these regions. Data are presented as means ± SEM. Statistical analysis was performed using 3-way ANOVA followed by Tukey's post-hoc test; ****p < 0.0001; n = 5 per group.

Additionally, ASIC1 protein expression in the SC and DRG of GlaKO animals exhibited a clear rostro-caudal gradient, with levels increasing toward the lumbar regions — approximately twofold higher than in cervical and thoracic segments — and further elevated at 8 compared to 4 months. This regional and age-dependent upregulation followed a consistent pattern across both tissues. For example, in 8-month-old GlaKO males, lumbar DRG levels reached 15.745 ± 0.307, compared to 6.829 ± 0.215 in the cervical DRG. A similar distribution was observed in the SC **(**Fig. 2**).**

Overall, these findings are consistent with our previous in vitro observations showing increased ASIC1 protein levels in neuronal cultures following Gb3 treatment (Salinas et al., 2020), as illustrated in Supplementary Fig. S1**C**. This supports the notion that neurons could respond to Gb3 with increased ASIC1 expression.

### Mechanical and thermal sensitivity in GlaKO animals over time

**Fig. 3:**
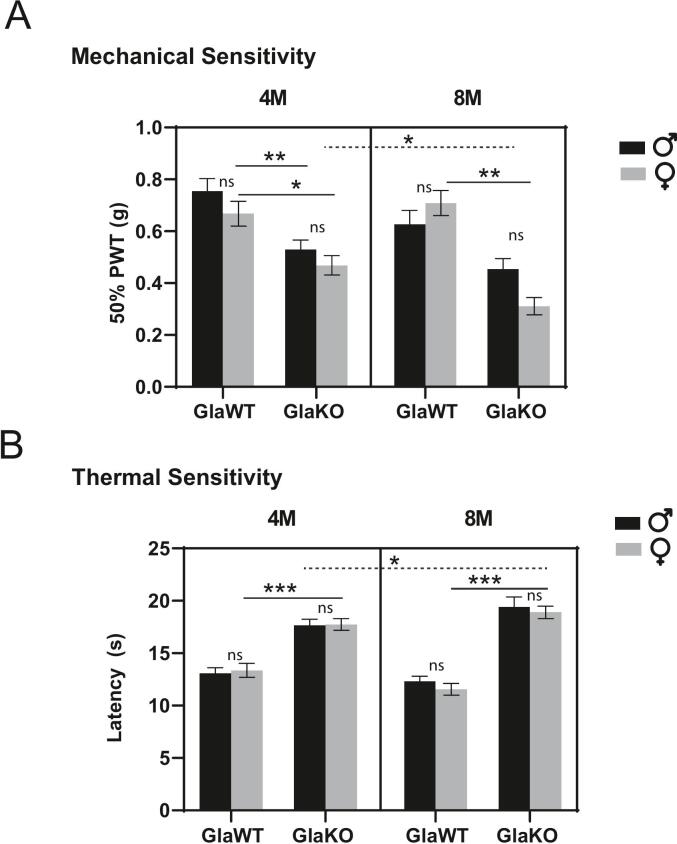
**Mechanical and thermal sensitivity of GlaKO mice(A)** Plot depicting mechanical sensitivity, measured as the 50 % paw withdrawal threshold (PWT) in grams, using the Von Frey test in 4- and 8-month-old male and female GlaKO and GlaWT mice. Note the increased sensitivity in GlaKO animals, which becomes more pronounced with age. Significant differences between male and female GlaKO animals become apparent at 8 months. **B)** Plot illustrating thermal sensitivity, measured as latency to respond in seconds, using the hot plate test. GlaKO animals exhibit hyposensitivity compared to GlaWT controls. Data are presented as means ± SEM; n = 11–30 per group. Statistical analysis was performed using a generalized linear model (GLM) with Log-Normal error structure and logarithmic link function for both mechanical and thermal sensitivity data, followed by Tukey's post-hoc test. ns = not significant; * p < 0.05; ** p < 0.01; *** p < 0.001; **** p < 0.0001.

Building on our previous findings, we aimed to determine whether the observed increase in ASIC1 channels could be pharmacologically modulated in a behavioral context. We conducted tests to assess sensory responses in the GlaKO mouse model, focusing on mechanical and thermal stimuli due to the established association with these sensations in FD. Our primary interest was in the response to mechanical stimulation, given its relevance to ASIC function. As shown in Fig. 3, our data confirm earlier reports that GlaKO animals exhibit mechanical hypersensitivity (Jabbarzadeh-Tabrizi et al., 2020).

Paw withdrawal threshold (PWT) values indicated a marked increase in mechanical sensitivity in GlaKO animals compared to controls. At 4 months, mean 50 % PWTs were 0.668 ± 0.048  g and 0.755 ± 0.048  g for GlaWT males and females, respectively, whereas GlaKO males and females exhibited significantly lower thresholds of 0.529 ± 0.037  g and 0.469 ± 0.038  g. This heightened sensitivity in GlaKO animals became more pronounced at 8 months, with PWTs further decreasing to 0.454 ± 0.041  g in males and 0.311 ± 0.034  g in females. Despite the overall genotype-dependent reduction in PWT, no significant sex-related differences were detected within each genotype.

In contrast, these animals displayed thermal hyposensitivity, which aligns with observations from other studies (Jabbarzadeh-Tabrizi et al., 2020)*.* Mean latency values in GlaWT animals were 13.094 ± 0.513  s for males and 13.370 ± 0.667  s for females, whereas GlaKO animals exhibited significantly longer latencies of 17.650 ± 0.590  s (males) and 17.730 ± 0.562  s (females). While thermal thresholds in GlaWT animals remained relatively stable at 8 months, GlaKO animals showed a further increase in latency, reaching 19.430 ± 0.920  s in males and 18.920 ± 0.598  s in females. No significant sex-related differences were detected within each genotype.

### Pharmacological modulation of ASICs and Mechanosensation

Given the hyposensitive response to thermal stimuli and the documented information on ASIC1 channels in mechanical sensitivity, we concentrated our pharmacological investigation on mechanical sensitivity. Specifically, we explored the impact of the ASIC1a blocker Psalmotoxin-1 (Pctx-1), administered intrathecally, following a protocol similar to that used in the formalin mouse model (Gobetto et al., 2022) to determine if the increased mechanical sensitivity could be modulated.

**Fig. 4:**
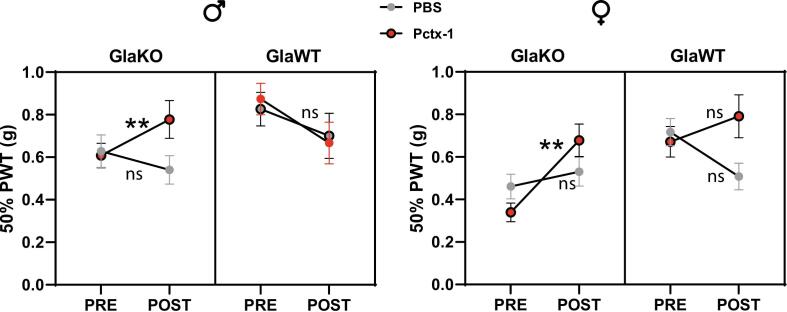
**Modulation of Mechanical sensitivity by Pctx-1 in GlaKO mice** Plot illustrating the Mean 50 % paw withdrawal threshold (PWT) in males (left) and female (right) groups of GlaKO and GlaWT mice before (PRE, filled dot) and after (POST, empty dot) intrathecal administration of PBS or Pctx-1. Pctx-1 treatment significantly increased the 50 % PWT, indicating a reduction in mechanical sensitivity in GlaKO animals. Statistical analysis was performed using a linear mixed-effects model including a constant variance function by treatment crossed with genotype to explain the heteroscedasticity. Tukey tests were used as post-hoc analysis: ns = not significant; **p < 0.01. Data are presented as means ± SEM. Lines connecting pre- and post-treatment average values are included to illustrate changes within each group and facilitate visual comparison between male and female plots. Group sizes: GlaKO males, PBS (n = 19), Pctx-1 (n = 14); GlaKO females, PBS (n = 21), Pctx-1 (n = 17); GlaWT males, PBS (n = 11), Pctx-1 (n = 12); GlaWT females, PBS (n = 12), Pctx-1 (n = 11). Additional details including SEM and tables with individual group means for pre- and post-treatment conditions are provided in Suppl. Fig. 2.

Our findings, shown in Fig. 4, demonstrate that Pctx-1 administration effectively reduced mechanical sensitivity in GlaKO animals. In males, sensitivity decreased as illustrated by PWT parameters increasing from 0.608 ± 0.058 to 0.777 ± 0.089 following Pctx-1, and in females, from 0.608 ± 0.058 to 0.777 ± 0.098. No significant differences were observed in GlaKO animals treated with PBS instead of Pctx-1, nor in GlaWT animals treated with either compound. Interestingly, while both GlaKO males and females responded to Pctx-1, the magnitude of the effect differed significantly between sexes. These results highlight the involvement of ASIC1a channels in mechanical sensation in this model (see Suppl. Fig. S2 for detailed values and statistical results by sex).

### Signaling via ASIC1a

We next explored the downstream signaling pathways associated with ASIC1a, with a focus on ERK kinases. Our aim was to determine whether the phosphorylation of ERK kinases, known to be involved in pain signaling, is influenced by ASIC1a activity in FD and whether this pathway is affected by the ASIC1a blocker, Psalmotoxin-1 (Pctx-1).

**Fig. 5:**
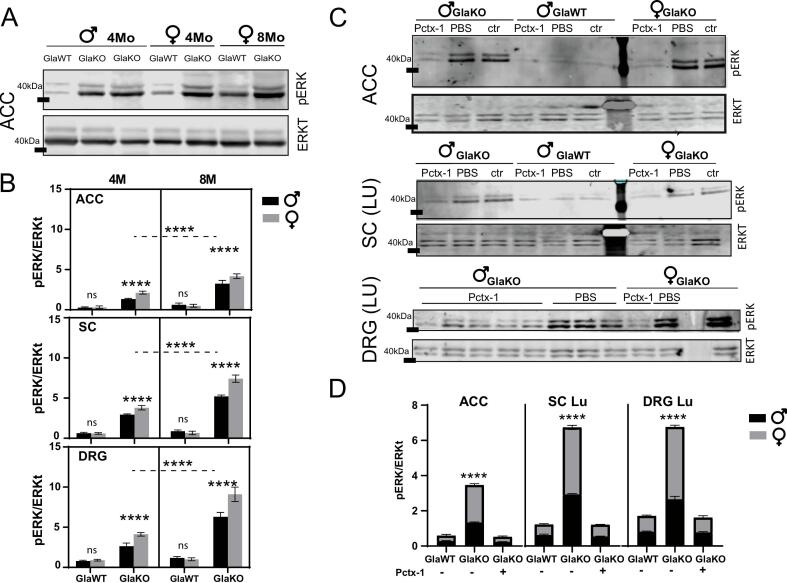
**ERK pathway modulation by Pctx-1A)** Representative Western blot (WB) membrane showing phospho-ERK (pERK) and total ERK (ERKt) levels in the anterior cingulate cortex (ACC) of 4- and 8-month-old male and female mice. Notice the increased pERK signal in GlaKO animals compared to GlaWT controls. **B)** Quantification of pERK/ERKt ratios in the ACC, lumbar spinal cord (Lu SC), and lumbar dorsal root ganglia (Lu DRG) of 4- and 8-month-old male and female GlaKO and GlaWT mice. **C)** Representative WB membranes illustrating pERK and ERKt levels in the ACC, Lu SC, and Lu DRG of 4-month-old male and female GlaKO and GlaWT mice, with comparisons between untreated controls (Ctr) and animals treated with Pctx-1 or vehicle (PBS) via intrathecal administration. Note that Pctx-1 treatment reduces pERK levels in GlaKO animals to those observed in GlaWT mice. **D)** Quantitative analysis of pERK/ERKt ratios from membranes like those shown in panel C, comparing GlaWT and Pctx-1-treated GlaKO mice across different regions. In all regions analyzed, Pctx-1 treatment in GlaKO animals restores pERK levels to those of GlaWT controls. Data are presented as means ± SEM; ****p < 0.0001, with comparisons made using 3-way ANOVA followed by Tukey’s post-hoc tests; n = 5 per group.

As shown in Fig. 5, we observed a significant increase in ERK kinase phosphorylation in GlaKO animals, both, male and females. In the ACC, pERK/total ERK (pERK/ERKt) ratios were markedly elevated in GlaKO animals, reaching 1.331 ± 0.041 in males and 2.130 ± 0.082 in females, compared to 0.276 ± 0.037 (males) and 0.316 (females) in GlaWT animals. A similar pattern was observed in the lumbar DRGs, where GlaWT animals showed pERK/ERKt values of 0.812 ± 0.039 (males) and 0.897 ± 0.046 (females), whereas GlaKO levels increased to 2.648 ± 0.174 (males) and 4.117 ± 0.099 (females). This trend was also evident in the lumbar SC. For full datasets and statistical details, refer to Supplementary Fig. S2.

The administration of Pctx-1 led to a marked reduction in ERK phosphorylation in the ACC, SC, and DRGs, suggesting that the observed decrease in mechanical sensitivity may be mediated, at least in part, through inhibition of this signaling pathway. This effect was consistently observed across the ACC, SC, and DRGs. Following Pctx-1 treatment, phosphorylation levels decreased to values comparable to those of GlaWT animals—for example, in the ACC, pERK/ERKt ratios reached 0.232 ± 0.017 in males and 0.293 ± 0.048 in females; in the lumbar DRGs, values were 0.771 ± 0.049 in males and 0.848 ± 0.101 in females. The differences in pERK/ERKt closely mirrored the pattern of ASIC1a upregulation. This increase in pERK levels could reflect a downstream effect of ASIC1a activity, as our previous in vitro studies showed that ASIC1a upregulation in neurons was accompanied by increased pERK levels. While these findings raise the possibility of ERK pathway involvement in ASIC1a-related signaling, further studies using cell-type-specific approaches will be required to clarify the origin and functional significance of pERK activation in Fabry disease.

### Regulatory mechanisms of ASIC1 protein expression in pain-related areas in a Fabry mouse model versus an acute pain model

**Fig. 6:**
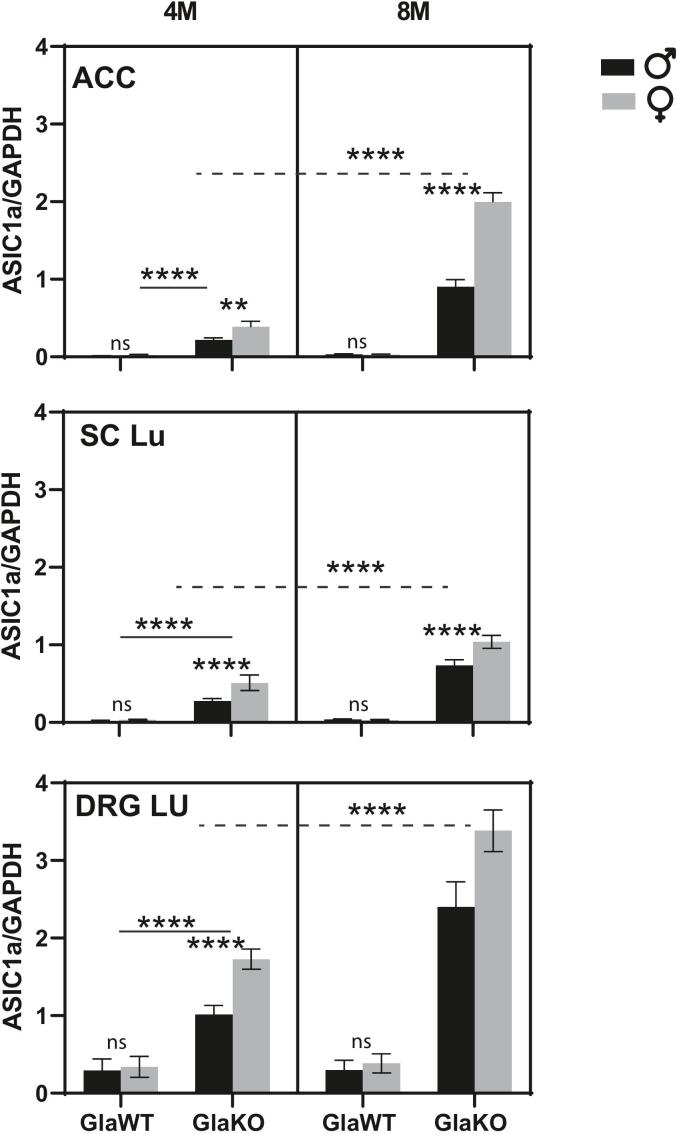
Plots showing ASIC1a mRNA levels normalized to GAPDH (housekeeping gene) in the anterior cingulate cortex (ACC), lumbar spinal cord (Lu SC), and lumbar dorsal root ganglia (Lu DRG) of 4- and 8-month-old male and female GlaKO and GlaWT mice. An increase in ASIC1a mRNA is observed in the analyzed areas of GlaKO mice compared to GlaWT controls. Data are presented as means ± SEM. Statistical significance was determined using 3-way ANOVA followed by Tukey’s post-hoc tests; ****p < 0.0001; n = 5 per group.

To further explore the mechanisms regulating ASIC1a protein expression, we determined ASIC1a mRNA levels in the analyzed tissues. In our previous study using the mouse formalin-induced acute pain model, we observed elevated ASIC1 protein levels in these tissues, which were not associated with increased mRNA levels, but with a decrease in microRNAs known to regulate ASIC1 protein levels, specifically miR-485-5p (Gobetto et al., 2022). In the current study, we focused on the chronic pain model of Fabry disease and assessed ASIC1a (the Pctx-1-responsive subunit) mRNA levels across the same tissues. Our results, shown in Fig. 6, reveal a consistent correlation between mRNA levels and protein expression across all tissues analyzed.

Our results, reveal a consistent correlation between mRNA levels and protein expression across all analyzed tissues. In the ACC, ASIC1a mRNA levels in GlaWT animals were 0.013 ± 0.001 (males) and 0.024 ± 0.003 (females), while GlaKO animals exhibited markedly higher levels: 0.218 ± 0.012 (males) and 0.389 ± 0.032 (females), which further increased at 8 months to 0.904 ± 0.040 and 1.997 ± 0.053, respectively. Similarly, in lumbar DRGs, GlaWT animals showed values of 0.294 ± 0.065 (males) and 0.340 ± 0.061 (females), whereas GlaKO levels rose to 1.015 ± 0.053 (males) and 1.729 ± 0.058 (females), reaching 2.402 ± 0.144 and 3.384 ± 0.120, respectively, at 8 months. We also measured miR-485-5p levels, finding that they remained unchanged, as depicted in Supplementary Fig. S2.

## Discussion

The findings of this study emphasize the relevance of ASIC1a in pain mechanisms in FD. Additionally, the possibility of modulating ASIC1a constituted channels by means of Pctx-1 support the development of ASIC1a-targeted therapies as a strategy in pain conditions, especially in diseases like Fabry disease where glycosphingolipid accumulation seems to contribute to increased ASIC1a protein expression.

### ASIC1 in acute and FD pain in central and peripheral regions

This study analyzed both central and peripheral nervous system regions to understand ASIC1a's role in pain modulation. Specifically, we focused on the anterior cingulate cortex (ACC) and spinal cord (SC) as central regions, and the dorsal root ganglia (DRGs) as key peripheral sites. Our data suggest that upregulation of ASIC1a at multiple levels of the pain neuraxis may contribute to pain hypersensitivity in acute inflammatory and chronic metabolic pain conditions.

In the central nervous system, the dorsal horn of the SC is a critical point for processing nociceptive input (Chafaï et al., 2023). ASIC1a, particularly in its homomeric form, has been proposed to contribute to windup mechanisms that lead to spinal facilitation and pain hypersensitivity (Duan et al., 2007, Chafaï et al., 2023). Through these processes, ASIC1a could play a role in the integration of nociceptive signals arriving from the periphery. Further upstream, in the ACC, ASIC1a has been shown to be essential for both thermal and mechanical sensitivity associated with chronic pain (Cao et al., 2009). The activation of the ERK pathway in the ACC has been linked to pain processes, and injections of Pctx-1 into the ACC prevent pain responses, confirming the importance of ASIC1a (Li et al., 2019). Our current findings extend previous observations of ASIC1a protein upregulation in the ACC in a formalin-induced acute pain model.

In the peripheral nervous system, we observed increased ASIC1a protein expression in DRGs in both the current study and our earlier formalin model work. This upregulation may reflect activation of ASIC1a channels by ongoing inflammatory signals — an effect consistent with the notion of an “inflammatory soup” (Rozenfeld and Feriozzi, 2017). Supporting this, Choi et al. found that subcutaneous administration of lysoGb3 in mouse paws resulted in mechanical allodynia, which was associated with increased calcium currents from voltage-activated calcium channels in small-diameter neurons of the IB4 nonbinding, peptidergic subclass, with a high percentage of these neurons also expressing TRPV1 channels (Choi et al., 2015). Thus, metabolic changes in FD appear to sensitize peripheral sensory neurons. Papalampropoulou-Tsiridou et al. studying a model of nerve injury rather than inflammation or metabolic disease, found no increase in ASIC1a mRNA in lumbar DRGs, but reported an upregulation of ASIC1b instead s (Papalampropoulou-Tsiridou et al., 2020). In contrast, under Fabry conditions, we observed an increase in both ASIC1 protein and ASIC1a mRNA in lumbar DRGs, along with elevated levels of phosphorylated ERK. This ERK activation was reduced by Pctx-1, a selective inhibitor of ASIC1a homotrimeric channels, supporting a mechanistic link between ASIC1a activity and ERK signaling in Fabry-associated pain. This may highlight distinct channel regulation mechanisms in different pain conditions. Our findings are particularly relevant to the lumbar regions which innervate the lower limbs where FD patients commonly experience pain. The observed ASIC1a upregulation in lumbar DRGs correlates with mechanical hypersensitivity in the hind paws of mice, suggesting enhanced nociceptive signaling in this region may underlie limb pain in FD. Parallel ASIC1a increases in the lumbar spinal cord further implicate both peripheral and central sites in FD pain mechanisms. The regional difference in ASIC1a expression between cervical/thoracic and lumbar segments raises the question of whether front and hind paws differ in mechanosensitivity. Our study focused on hind paw responses, which are standard in nociceptive tests due to lower variability, but front paw analysis could provide complementary insights. Given the lack of ASIC1a upregulation in cervical DRGs, it would be informative to assess whether front paw sensitivity is similarly unaffected. While grooming-related use of forepaws may introduce behavioral variability, future studies including front paw assessments could help clarify region-specific roles of ASIC1a in FD.

While this study did not directly address the mechanism or cellular origin of the increased ASIC1a expression in vivo, our previous *in vitro* work in Fabry disease models (Salinas et al., 2020) showed that Gb3 and its derivative lyso-Gb3, as well as pharmacological inhibition of GLA, led to elevated levels of ASIC1a mRNA and protein in HEK cells and primary mouse cortical and hippocampal neurons. Although the precise pathways remain to be fully determined, inflammatory signaling cascades, such as NF-κB activation and increased cytokine levels (e.g., IL-1β, IL-6), have been implicated in the regulation of ASIC1a under pathological conditions. Given that GLA deficiency has been associated with neuroinflammatory processes and altered lipid signaling, these pathways could represent candidate mechanisms linking GLA dysfunction and ASIC1a upregulation. Further work will also be required to identify the specific cell types contributing to this upregulation *in vivo*.

Comparing the acute formalin and FD models reveals an increase in ASIC1a protein levels in DRG neurons, indicating that ASIC1a upregulation may be a common feature of pain pathways. This aligns with previous reports of increased ASIC1a transcript levels in DRGs in various models of persistent inflammatory or neuropathic pain. However, important differences between the models suggest distinct underlying mechanisms. In the formalin model (Gobetto et al., 2022), ASIC1a protein increased without corresponding changes in mRNA levels, consistent with a post-transcriptional regulation mediated by microRNAs. In contrast, our FD model showed increases in both mRNA and protein levels of ASIC1a, suggesting transcriptional upregulation. These differences may reflect distinct regulatory mechanisms, possibly influenced by the transient nature of formalin-induced inflammation versus the persistent pathological context in FD. Moreover, while the formalin model reflects an acute inflammatory insult, the FD model is characterized by a chronic lysosomal dysfunction with progressive glycosphingolipid accumulation and neuroinflammatory changes. Thus, despite shared outcomes at the level of ASIC1a, the FD model captures a broader disease-relevant context and provides novel insights into ASIC1a involvement in genetically driven pain conditions. Further studies are required to establish its specific contribution to chronic pain mechanisms in broader pain models. Nonetheless, the data highlight ASIC1a as a potential contributor to pain pathophysiology in FD, warranting future investigation into its role in sustained pain states.

### ASIC1-related mechanical and thermal sensitivity in FD mice

Different ion channels have been analyzed related to FD (Weissmann et al., 2021, Hofmann et al., 2018, Lakomá et al., 2014, Choi et al., 2015, Hofmann et al., 2024) and sensory alterations. Mechanical hypersensitivity is a major symptom of neuropathic pain. FD patients show both thermal and mechanical alterations. In our study, we detected hypersensitivity to mechanical stimuli and hyposensitivity to thermal stimuli. This aligns with sensory profiles reported by patients, as well as findings from some FD models (Formaggio et al., 2022). Duan et al. described spinal circuits, including dynorphin neurons, as part of a mechanism that can gate mechanical pain—normally inhibited—without affecting thermal or cold sensation (Duan et al., 2015). Supporting the involvement of mechanosensory pathways, Jabbarzadeh-Tabriz et al. reported Gb3 accumulation in mechanoreceptive DRG neurons in the GlaKO model, but not in controls (Jabbarzadeh-Tabrizi et al., 2020), suggesting a possible structural substrate for altered mechanical pain signaling in FD (Jabbarzadeh-Tabrizi et al., 2020).

We focused on the mechanical hypersensitivity observed in mice, which relates to mechanical sensory alterations that have been associated with ASIC1a channels. Although our pharmacological intervention specifically targeted mechanosensory responses, we also assessed thermal sensitivity and found significant alterations consistent with previous reports in Fabry disease models. However, the effect of ASIC1a blockade on thermal sensitivity was not evaluated in this study. Thus, while our findings support a role for ASIC1a in mechanosensory alterations, its contribution to thermal hypersensitivity remains to be determined. Future studies will be necessary to evaluate whether ASIC1a also modulates thermal nociception in this model, which would help clarify the modality-specific involvement of this channel in Fabry-related pain. In this context, although we did not assess TRPV1 in the present work, future investigations into its potential interaction with ASIC1a in sensory neurons could further elucidate the mechanisms underlying both thermal and mechanical hypersensitivity. This is particularly relevant given the established contribution of both channels to pain signaling under acidic or inflammatory conditions, such as those observed in FD, and the reported association of TRPV1 with thermal sensitivity in this disease (Lakomá et al., 2016).

### Sex and age differences in FD mice

The distribution of ASIC1 channels has been documented across various brain regions, neurons, and glia (Wemmie et al., 2003, Zha, 2013, Yu et al., 2015 Mar, Wang et al., 2022). Throughout development in mice from embryonic to postnatal stages, Alvarez de la Rosa et al. reported that ASIC1a protein expression remains relatively stable from early embryonic stages (E12) through adulthood (Alvarez de la Rosa et al., 2003). In the medulla, studies have shown a decrease in ASIC1 protein and ASIC1a mRNA expression in adult rats compared to neonatal rats (Song et al., 2016). Similarly, in cultured cortical neurons, maturation from day 7 to day 28 led to changes in the ratio of ASIC1a/ASIC2a mRNA expression, accompanied by a reduced sensitivity to PcTX-1, suggesting a lower expression of homomeric ASIC1a channels (Li et al., 2010). This data agree with our findings showing expression at 4 and 8 month old mice were not altered. However, in FD mice differences were observed based on both, sex and age.

Uçeyler et al. reported hypersensitivity to mechanical pain in GlaKO mice, independent of sex (Üçeyler et al., 2016). However, other studies indicate that sex-related differences do exist. For example, Rullo et al. found pain sensitivity alterations in male but not female GlaKO mice (Rullo et al., 2021) highlighting the variability in responses depending on the experimental model and conditions.

In GlaKO mice, in this study, ASIC1a protein expression was significantly higher in females than in males, whereas no sex-related differences were found in control animals. Despite this, both sexes exhibited similar mechanical and thermal sensitivity at 4 months of age (Fig. 3), suggesting that elevated ASIC1a protein levels in females did not translate into baseline behavioral differences (Fig. 1, Fig. 2). Interestingly, Pctx-1 administration revealed a sex-dependent effect, with females showing a greater response (Fig. 4). This suggests that while ASIC1a protein is more abundant in females, its functional impact may be modulated by compensatory mechanisms or differential activation thresholds. Pctx-1 may have unmasked these differences, highlighting a latent contribution of ASIC1a to sensory processing in females. Future studies will focus on elucidating the mechanisms underlying this sex-specific modulation, including potential roles of ASIC1a trafficking, activation, and hormonal regulation.

Regarding age, Rodrigues et al. further demonstrated that Gb3 accumulation in GlaKO mice increases with age in various regions, including the brain, potentially contributing to the sensory abnormalities observed (Rodrigues et al., 2009).

Studies in FD patients also show a complex relationship between age, sex, and pain sensitivity in Fabry disease. Liao et al. reported a bell-shaped distribution of pain scores in male patients, peaking between the early 20 s and late 40 s. However, in female patients, the association between pain and age was more variable (Liao et al., 2022). This variability could be explained by a reduction in intraepidermal nerve fiber density (IENFD) with age, as documented by Uçeyler (Üçeyler et al., 2011) which may influence sensory abnormalities in FD.

Additionally, in this study, while biochemical differences were observed between male and female GlaKO mice, these did not always translate into significant behavioral changes, particularly at younger ages. By 8 months, the differences in ASIC1a protein expression between male and female mice became more apparent, with females showing strikingly higher levels of expression. This raises the question of whether a certain threshold of ASIC1a channels must be reached before these biochemical differences translate into behavioral changes or whether specific circumstances, such as pain triggers, are required to reveal these changes.

Moreover, inflammatory pathways may play a role in the heightened pain sensitivity observed in females. Hofmann et al analyzed differences between male and female patientś fibroblasts and found the cytokine IL-8 expression increased in females, thus proposing IL-8 as a contributor to pain in female FD (Hofmann et al., 2024).

We have previously analyzed ASIC1 channels in the same regions of male and female C57B/L6 mice, documenting no sex differences under baseline conditions. However, following exposure to a noxious stimulus (formalin), an increase in ASIC1 channels was observed in both male and female animals, with females showing significantly higher expression than males in the ACC (Gobetto et al., 2022). The role of different brain regions in contributing to these sex differences warrants further investigation.

Although male Fabry patients often present with more severe pain clinically, our finding of increased ASIC1a protein expression in female GlaKO mice highlights important molecular sex differences that may not directly translate into clinical pain severity. Our behavioral assessments showed similar baseline nociceptive sensitivity between sexes, suggesting that increased ASIC1a levels in females might be modulated by compensatory mechanisms or require specific triggers to impact pain sensitivity. This complexity reflects the heterogeneous clinical pain phenotypes in female patients, which are influenced by multiple factors including age, hormonal status, and inflammation. Future studies could investigate the role of heterozygous females to further clarify sex-specific mechanisms.

### Mouse model considerations

Mouse models of Fabry disease vary in their breeding strategies and genetic backgrounds, which can influence phenotypic outcomes and experimental interpretation. A potential limitation of this study is the absence of littermate controls. Breeding strategies are not always explicitly detailed in the literature, making it difficult to assess the potential influence of this type of variability. Notably, Jabbarzadeh‑Tabrizi et al. (Jabbarzadeh-Tabrizi et al. 2020) compared Fabry disease mouse models maintained on different genetic backgrounds—C57BL/6 versus a mixed B6/129 strain—and demonstrated that the latter exhibited earlier onset of cardiac and renal hypertrophy as well as more pronounced sensory deficits. These findings highlight how background strain can significantly influence disease phenotype. In the present study, we also used the mixed B6/129 background, with separate homozygous GlaWT and GlaKO colonies to ensure consistent genetic and environmental conditions across experimental groups. This strategy is particularly suited for X-linked genes such as Gla (additional considerations related to breeding design are detailed in the Materials and Methods section).

Altogether, while each breeding strategy has its strengths and limitations, confirming these findings across diverse Fabry disease models in future studies would provide stronger mechanistic support and enhance their translational relevance.

### Therapies using ASIC1a blockers

ASIC1a blockers have gained increasing attention as potential therapeutic agents. In ischemic events, various studies have explored the use of different drugs, and even analyzed factors such as the therapeutic time window, drug concentration, and the ability of molecules to cross the blood–brain barrier (Pignataro et al., 2007, Xiong et al., 2004). These blockers have also been investigated in models of neurodegenerative diseases, showing potential for broader applications (Mango and Nisticò, 2021).

In the context of pain, one pathway that has been extensively studied is the ERK signaling pathway. However, previous attempts to block the ERK kinase pathway in therapeutic interventions have faced challenges, including cognitive side effects (Zhen et al., 2023). A more targeted approach, selectively focusing on pain-related ERK activation, could present new therapeutic opportunities.

We have given evidence for a role of ASIC1a in modulating pain through this pathway. Thus, selectively targeting ASIC1a to influence ERK activation could lead to new pain therapies. Moreover, our findings suggest that both sex and age could significantly impact the effectiveness of ASIC1a-targeted therapies. This points to the importance of personalized treatment strategies, particularly in conditions like FD, where these factors may influence therapeutic outcomes.

## CRediT authorship contribution statement

**Mayra Micaela Montes:** Methodology, Investigation, Formal analysis. **Libia Catalina Salinas Castellanos:** Methodology, Investigation, Formal analysis, Data curation. **Georgina Oriana Mingolo Malnati:** Investigation. **Juan Santiago Guidobono:** Formal analysis, Data curation. **Ariel Félix Gualtieri:** Data curation. **Mariela Lacave:** Investigation. **Romina De Lucca:** Resources, Investigation. **María Natalia Gobetto:** Investigation. **Pablo Gabriel Vetta:** Resources, Methodology. **Zaira Soledad Verónica Náguila:** Methodology. **Fernanda Toledo:** Methodology. **Osvaldo Daniel Uchitel:** Resources, Funding acquisition, Conceptualization. **Carina Weissmann:** Writing – review & editing, Writing – original draft, Supervision, Resources, Methodology, Investigation, Funding acquisition, Formal analysis, Data curation, Conceptualization.

## Declaration of competing interest

The authors declare that they have no known competing financial interests or personal relationships that could have appeared to influence the work reported in this paper.
