# Supplementary material for: ASIC1a-associated mechanical hypersensitivity in the GlaKO Fabry disease mouse model
Source: Neurobiol Pain. 2025 Jun 26;18:100189. doi: 10.1016/j.ynpai.2025.100189 (PMC12270731; doi:10.1016/j.ynpai.2025.100189)
Supplement: Supplementary Data 1 [file mmc1.docx]

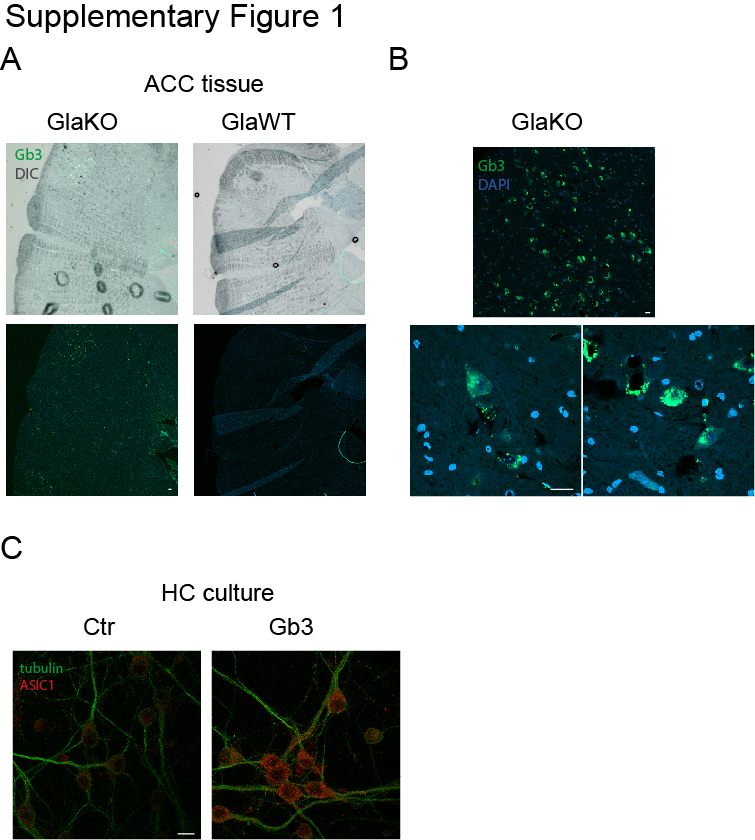
 **Supplementary Figure 1 A)** Representative micrographs of anterior cingulate cortex (ACC) sections from perfused GlaKO and GlaWT mice, stained for Gb3 (green, upper panels). The corresponding merged images with brightfield microscopy (lower panels) are shown to illustrate the anatomical region at low magnification (4× objective).**B)** High-magnification confocal images from 4-month-old male GlaKO mice stained for Gb3 (green) and counterstained with DAPI (blue) to visualize nuclei. Images were captured using 40× (left) and 60× (middle and right) objectives to highlight cellular localization of Gb3. Scale bar = 10 µm. **C)** Representative images from primary hippocampal cultures (HC) derived from C57BL/6 mouse embryos, either untreated (Ctr) or treated with Gb3 (Gb3), and stained for ASIC1 (red) and tubulin (green). An increase in ASIC1 signal is evident in Gb3-treated cultures. 40× objective; scale bar = 10 µm. Culture and staining protocols were performed as previously described (Salinas et al., 2020 (17)**.**


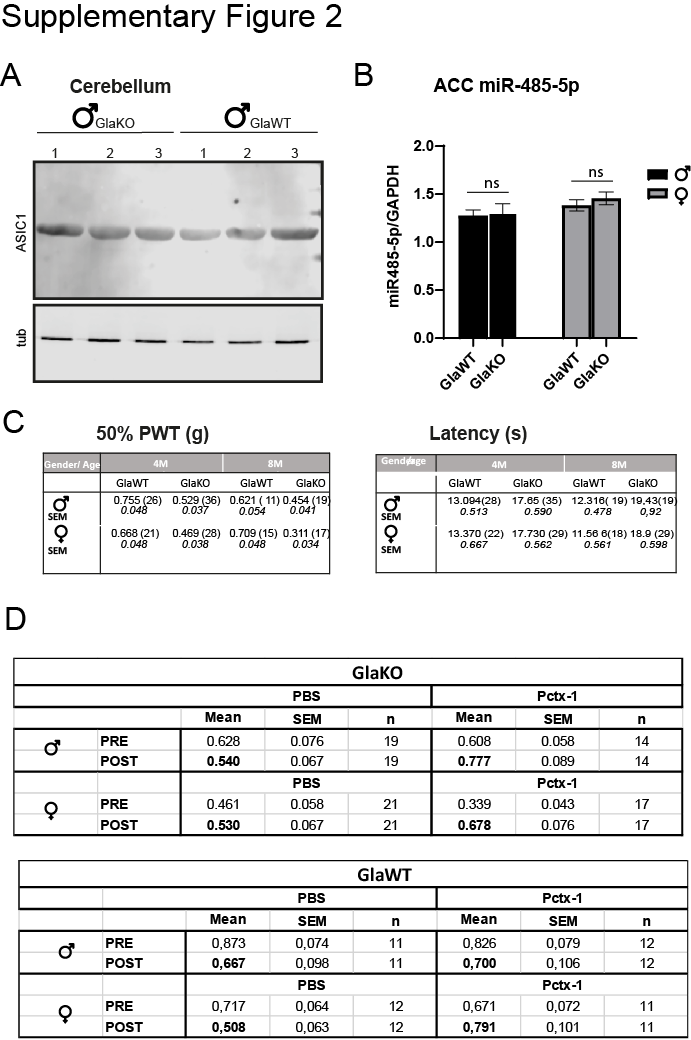


**Supplementary Figure 2** **A)** Western blot analysis of ASIC1 proteins in cerebellar lysates from GlaKO and GlaWT mice. Samples from three individual animals per group were analyzed. Tubulin was included as a loading control (lower membrane). No significant differences (ns) were observed between GlaKO and GlaWT animals. Male mice were used. **B)** Quantification of miR-485-5p levels, normalized to GAPDH, from mRNA extracted from GlaWT and GlaKO male and female mice. The plot indicates no significant differences between groups for either sex. Statistical analysis was performed using a Student's t-test; three animals were used per condition. **C)** Tables showing the 50% paw withdrawal threshold (PWT) in grams (left) and latency in seconds (right) for 4- and 8-month-old GlaWT and GlaKO male and female animals. Values are presented as mean ± SEM (standard error of the mean), with sample size (n) indicated in brackets. These data correspond to the parameters plotted in Figure 3. **D)** Tables showing the 50% paw withdrawal threshold (PWT) in grams in male (top) and female (bottom) mice following intrathecal administration of PcTX-1 or vehicle (PBS). Values are presented as mean ± SEM (standard error of the mean), with sample size indicated. The data correspond to the parameters plotted in Figure 4. Refer to the text for further details.


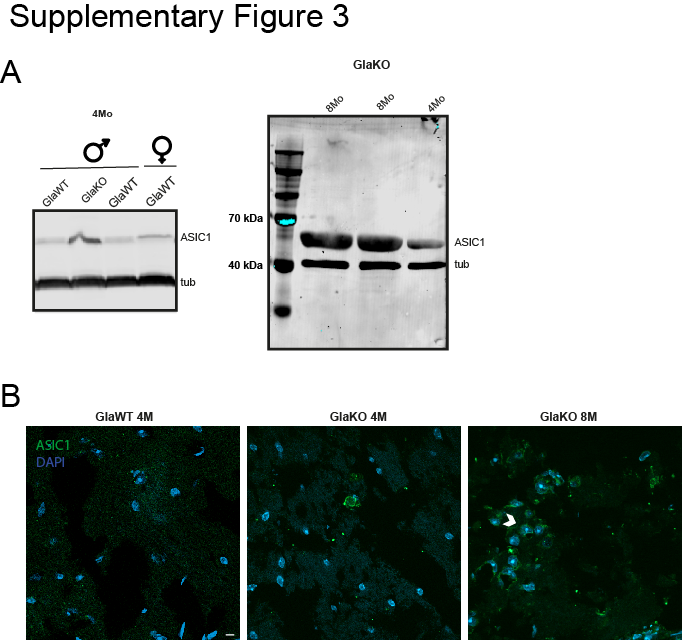


**Supplementary Figure 3 A)** Representative Western blot membranes showing equal protein loading (20 μg per lane) across conditions. Left: Lysates from 4-month-old GlaWT and GlaKO mice, separated by sex. Right: Lysates from GlaKO mice at 4 and 8 months of age. Tubulin band intensity and size remain consistent across samples, confirming equal loading. Variability in ASIC1 band intensity does not reflect loading differences. **B)** Representative immunostaining of the anterior cingulate cortex (ACC) from a 4-month-old GlaWT mouse (left), a 4-month-old GlaKO mouse (middle), and an 8-month-old GlaKO mouse (right), stained for ASIC1 (green) and counterstained with DAPI (blue). Although staining quality is suboptimal, an increase in ASIC1 signal (green) is noticeable in the 8-month-old GlaKO mouse (white arrowhead), consistent with Western blot findings. Scale bar: 10 μm.
